# The Salt Content of Lunch Meals Eaten at Danish Worksites

**DOI:** 10.3390/nu10101367

**Published:** 2018-09-24

**Authors:** Anne D. Lassen, Ellen Trolle, Anette Bysted, Pia Knuthsen, Elisabeth W. Andersen

**Affiliations:** 1 Division of Risk Assessment and Nutrition, National Food Institute, Technical University of Denmark, DK-2800 Lyngby, Denmark; eltr@food.dtu.dk (E.T.); pknuan@gmail.com (P.K.); 2 Research Group for Bioactives – Analysis and Application, National Food Institute, Technical University of Denmark, DK-2800 Lyngby, Denmark; anby@food.dtu.dk; 3 Danish Cancer Society Research Center, Statistics and Pharmacoepidemiology, DK-2100 Copenhagen, Denmark; elian@cancer.dk

**Keywords:** sodium, worksite canteen meals, salt reduction strategies, food and nutritional environment

## Abstract

Monitoring levels of sodium (salt) in meals consumed out-of-home is needed to support effective implementation of salt-reduction strategies. The objective of the study was to examine lunch salt intake at 15 worksite canteens and to compare with results from a comparable study conducted 10 years before. A duplicate-portion-technique with subsequent chemical analysis was used to quantify 240 customers’ lunch salt intake. Estimated mean salt intake was 2.6 g/meal (95% Cl: 2.2 to 3.0 g/meal) and 0.78 g/100 g (95% Cl: 0.69 to 0.88 g/100 g). Salt intake measured both as g per meal and per 100 g was found to be significantly higher for male compared with female participants (+0.10 g/100 g, 95% Cl: +0.02 to +0.17 g/100 g, *p* = 0.011). Compared with the study conducted 10 years before, there was a significantly lower estimated salt intake of 0.5 g/meal (95% CI: −0.8 to −0.2 g/meal, *p* = 0.001), suggesting a possible reduction in canteen lunch salt intake during a 10-year period. Still, 40% of the meals exceeded the Nordic Keyhole label requirements of maximum 0.8 g salt per 100 g for ready meals. A further reduction of salt intake is warranted to comply with salt reduction targets.

## 1. Introduction

Excessive sodium (salt) intake is associated with several adverse health events, particularly hypertension, a leading risk factor for death and disease burden worldwide [1,2,3,4]. The vast majority of evidence confirms the need to reduce the salt consumption especially among the population groups with the highest intake of salt [5,6,7]. In Denmark it is estimated that almost a quarter of the population consume 11 g salt or more per day [8]. A meta-analysis involving worldwide studies indicates an overall average salt intake of 9.3 g per day [9], i.e., well above the recommended level of less than 6 g per day for the general population [1,10]. Accordingly, a 30% relative reduction in mean population intake of salt is among the 10 global targets in the Global Action Plan 2013–2020 set by the WHO to prevent non-communicable diseases [11].

In developed countries, up to three-quarters of the total salt intake comes from food consumed out of home and processed foods, whereas salt added to food at the table and in home food preparation generally is a minor contributor to total sodium intake [12,13]. Thus, there is a great demand for food business operators to reduce the salt content in their products. Food product reformulation has been suggested to be a highly cost-effective strategy to reduce sodium intake of the population for both affluent and disadvantaged communities [14,15,16]. By reducing the amount of salt in foods, manufacturers and food-service operators can give consumers more control over their sodium intake [17].

In Denmark, salt reduction in processed food is stimulated by the Danish Salt Partnership—a public private partnership with governmental, industry and NGO involvement—since 2011 and by the Nordic Keyhole nutritional label on prepacked products since 2009. In 2012, the Danish Veterinary and Food Administration further introduced the possibility of labelling of freshly prepared meals with the Keyhole label for use in the restaurant and canteen sector. An important feature of the Keyhole certification process has been to increase the restaurant professionals’ awareness and knowledge about how to cook and serve healthy meals, i.e., provide restaurants with training and tools to be successful in their work with healthy meals, which included guidance on reducing the use of salt and salty foods [18]. In 2015, a new statutory order on the use of the Keyhole label was launched lowering the maximum limits for the content of salt in ready meals from 1.0 to 0.8 g of salt per 100 g, and not more than 3.5 g salt per meal in total [19]. This is part of a strategy to gradually lower the salt levels across the food supply. In 2017 a new The Danish Meal Label was created to provide guidelines to professional kitchens, e.g., at worksites, in serving nutritious food. A canteen kitchen can use the Danish Meal Label if it adheres to the principles of the label and reduces the amount of salt in the food [20].

Odom et al. suggest from a cross-sectional analysis that there is substantial agreement and support for actions among consumers to limit salt in commercially-processed and prepared foods including actions to lower salt in foods served in worksites [21]. WHO stresses the importance of monitoring by publishing baseline levels of sodium in foods and providing regular updates to track any changes in the sodium content [22]. This information can be used to gain support for salt reduction, maintain pressure on the food industry and evaluate the effectiveness of salt-reduction initiatives.

The objective of the present study was to monitor employees’ lunch salt intake at 15 Danish worksite canteens and to investigate gender differences in employees’ canteen food salt intake. A further aim was to compare with results from a comparable study conducted 10 years before.

## 2. Materials and Methods

Data collection took place between February and November in 2014. The study was performed in accordance with the ethical standards of the Helsinki Declaration of 1975, as revised in 2008.

The 15 worksite canteens participating in a study conducted 10 years before [23] were contacted and asked to participate in the present study. The 15 worksite canteens had been extracted at random from a central national register representing both city and provincial towns as described in a previous paper by Lassen et al. [23]. A total of eight of the 15 worksites agreed to participate. Two worksites did not exist anymore, two worksites did not have a canteen anymore and three worksites declined to participate due to lack of time. As replacements, seven new worksites were contacted to participate in the study. One of the contacted canteens declined to participate and a new one with a matching profile was contacted instead. The new worksites were recruited in the same geographical area to match the missing worksites in terms of size and occupation profile, i.e., five private financial and service organizations, eight private manufacturing and distribution organizations and two public sector organizations in both studies.

The duplicate-portion technique with subsequent chemical analysis was used to get valid estimates of employees’ salt intake at lunch. A slightly modified method of the duplicate-portion technique [24] was used where a laboratory technician collected the duplicate plates, instead of participants collecting their own duplicate plates, in order to reduce respondent burden and to disrupt the food selection and eating pattern as little as possible. A total of 240 duplicate portions of employees’ lunch meal selections were collected over 2 days from the 15 participating canteens (a total of 16 lunch meals at each canteen).

Employees were recruited at random by asking them at specific pre-decided time-points before they selected their meals in the dining area whether they would participate in the study. A laboratory technician then followed the participants and collected identical duplicate portions of the employees’ meal selections from the buffet. A maximum of two persons at each canteen rejected to participate. Both the originals and the duplicate portions were photographed to document that portion sizes were similar between the duplicate meals and the original meals. After the employees finished eating their lunch meals, they were asked to return the plates to the technicians to record food not consumed, including non-edible items and edible waste. The amount of food not consumed was then removed from the collected duplicate meal portions to be able to determine the actual salt intake. Finally, the duplicate meal portions were individually mixed and homogenized for analysis. Beverages were not included in the analysis.

The participating employees completed a short questionnaire while photos were taken of their meals (before eating their meals) to provide background information on gender, age, weight, height and occupation (i.e., skilled, unskilled worker, office worker and trainee and other). Body mass index was calculated from self-reported height and weight data. Kitchen background information including the meal serving system, number of daily canteen lunch meals served and overall occupation profile of the customers at the worksite according to gender distribution and proportion of customers with sedentary job functions was collected through structured interviews with the canteen managers.

The sodium content was measured by ICP-OES (Inductively Coupled Plasma – Optical Emission Spectrometry, PerkinElmer, Waltham, MA, USA) after nitric acid digestion by microwaves based on the principles in Ikem et al. [25]. The method was accredited by the Danish Accreditation Authorities (DANAK) according to ISO 17025. The salt intake was estimated from sodium content by multiplying with 2.5 [26]. All data on salt intake was calculated as g per meal (portion), g per 100 g and energy adjusted as g per 10 MJ. Energy intake was calculated from analysed content of dry matter, protein, fat and ash as described elsewhere [1,27].

The same procedure was applied at the study conducted 10 years before compared to the present study, except that salt intake was originally calculated from analysis of chloride content [27]. We recalculated these data based on existing analysis of sodium content to be able to compare results with the present study. Further, whereas 240 duplicate portions of employees’ lunch meal selections were collected in the present study only 180 duplicate portions at 15 worksite canteens were collected at the study conducted 10 years before [23]. Data in the present study was collected the same time of the year compared to the study conducted 10 years before, i.e., seven worksites were visited from February to June and eight worksites from August to November in both studies.

Linear mixed models were used to analyze data statistically. When comparing results according to gender, the analyses were adjusted for age group, body mass index (BMI) group, and occupation, and worksite canteen was taken into account as a random effect. The outcomes were transformed using Box–Cox transformations as this was reasonable when looking at the residual plots and then the estimates and effects were back-transformed to the original scale. When comparing results from the present study with results from the study conducted 10 years before adjustments for gender, age, BMI and occupation were taken into account and worksite canteen was added as a random effect. The model fit was checked by residual plots and QQ plots, and if necessary, Box–Cox transformation for normal distribution was performed. The estimated effects were back-transformed to the original scale and can be viewed as covariate-adjusted differences in medians. Differences in the characteristics of the study participants between the two studies were analyzed using chi-squared tests for grouped variables or Fisher’s exact test if the expected number in a cell was less than 5. For continuous variables, results were compared using *t*-tests with unequal variances.

## 3. Results

### 3.1. Characteristics of Worksite Canteens and Participants

Table 1 shows the characteristics of the participating canteens. The customers at the canteens were reported to be mainly males by 60% of the canteens, and to have mainly sedentary job functions by 67%. A total of 33% of the canteens were outsourced to an external catering company. The outsourced canteens represented a total of three different catering companies. At all worksites except for one, the food was prepared in-house.

Table 2 shows the demographic characteristics of the participants in the study. A total of 59% of the participants were men, 58% of the participants were 40 years or above, 39% of the participants had a BMI above 25 kg/m^2^ and 68% of the participants categorized themselves as office workers. All worksite canteen served buffet-style, i.e., customers directly view and select the different dishes they wish to consume, and decide how much food they take.

### 3.2. Canteen Lunch Salt Intake

Table 3 shows the unadjusted mean as well as the estimated mean lunch intake of salt. Estimated mean salt intake was 2.6 g/meal (95% Cl: 2.2 to 3.0 g/100 g), 0.78 g/100 g (95% Cl: 0.69 to 0.88 g/100 g) and 12.0 g/10 MJ (95% Cl: 10.6 to 13.6 g/10 MJ). In total, 40% of the meals exceeded the standard of 0.8 g/100 g set by the Keyhole label program [17].

When comparing estimated female and male participants’ intake, the mean intake of total salt per meal for the male participants was significantly larger than for the female participants (difference 0.8 g, 95% Cl: 0.4 to 1.1 g, *p* < 0.001) (Table 4). Also, salt intake expressed as g per 100 g was significantly higher for the male participants compared with the meals from female participants (difference 0.10 g/100 g, 95% Cl: 0.02 to 0.17 g/100 g, *p* = 0.011). In total, 29% of the meals consumed by the female participants and 48% of the meals consumed by the male participants exceeded the limit of the standard of 0.8 g per 100 g set by the Keyhole label program [17]. There was no significant difference between genders when salt intake was expressed as g per 10 MJ.

### 3.3. Comparison of Canteen Lunch Meal Salt Intake with a Study Conducted 10 Years Before

When comparing the present data with results from the study conducted 10 years before a significant decrease in salt intake was observed with regard to salt intake expressed as both g per 100 g (difference −0.16 g/100 g, 95% CI: −0.23 to −0.08 g/100 g, *p* < 0.001) and g/meal (difference −0.5 g/meal, 95% CI: −0.8 to −0.2 g/meal, *p* = 0.001), equal to a 16% reduction. The difference was only borderline significant when salt intake was expressed as g per 10 MJ (difference −1.1 g/10 MJ, 95% CI: −2.2 to 0.0 g/10 MJ, *p* = 0.056). No significant differences in participants’ profile were found between the two studies with regard to sex distribution, age, BMI or occupation. In the study conducted 10 years before, a total of 61% of the participants were males, 51% of the participants were 40 years or above, 39% of the participants had a BMI above 25 kg/m^2^ and 62% of the participants were categorized as office workers.

## 4. Discussion

Protecting and promoting health in the worksite setting, including reducing salt in the meals, is critical [22]. The results from the present study showed that the estimated mean lunch salt intake at 15 Danish worksite canteens was 2.6 per meal or 0.77 g per 100 g. Further, a difference between genders with regard to both total salt content per meal and per 100 g was seen in the present study. For comparison, a recent study exploring the salt content of meals served in seven university canteens in Portugal showed that meals (soup and main dish) on average had a salt content of 2.8 g of salt per serving [28]. With regard to gender differences in salt intake, a similar pattern was found by Rasmussen et al. [27], indicating slightly different food choice patterns between genders.

Results from the present study also suggest a possible reduction in mean lunch salt intake at Danish worksite canteens by half a gram per meal corresponding to a 16% reduction over a 10 years period. The same trend was seen when salt intake was expressed as g salt per 100 g. Still, 40% of the meals exceeded the current Nordic Keyhole label of maximum 0.8 g salt per 100 g for ready meals suggesting a need for further reduction of salt content in the canteen food. The UK salt reduction targets for 2017 for ready meals was 0.63 g salt per 100 g on average, i.e., close to 20% lower compared to results found in the present study [29].

The need for further reduction of salt in canteen meals is supported by the relatively high salt density (salt intake compared to energy intake), although, a borderline significant reduction in salt density was seen across time. If the mean amount of salt in the lunch meal in the present study is extrapolated to a daily intake, it would be 12 g per day for a person consuming 10 MJ. This is a high value compared to the recommended long-term goal of 6 g per day. The extrapolation assumes that the salt content per MJ is similar in all meals consumed during the day [27]. This does not have to be true. Dickinson et al. found sodium density to be highest at lunch among participants of a nationally representative survey in Australia [30]. In the Healthy Eating Index-2015, a sodium density of 2.0 mg/kcal (corresponding to 12 g salt/10 MJ) or higher receives minimum points for the sodium component of the index [31]. For comparison, a total of 63% of the lunch meals in the present study exceeded this level. Zang et al. found from a cross-sectional study that although eating at restaurants was significantly related to higher intake of dietary sodium, no significant association was observed between eating at company/school canteens and higher daily dietary sodium intake [32]. This was suggested to be a result of a reduction in salt content due to government departments engaging canteens in nutritional dietary interventions, such as the promotion of nutrition and health knowledge and nutrition training for employees [33].

The reduction in the canteen lunch intake found in the present study might likewise be a result of several factors, including governmental initiatives such as the possibility of labelling of freshly prepared meals and establishing of a Salt Partnership, resulting in increased awareness and knowledge among restaurant professionals on how to cook and serve healthy meals and limiting the use of salt and salty products. Another possible factor is a change in major food group selections, e.g., an increase in fruit and vegetable intake, which might “dilute” the salt concentration of the meals. This might be a result of both the canteen priorities in composition of the meals, a shift toward more buffet-style serving and the preferences of the employees, especially with regard to increased preference to fruits and vegetables. Rasmussen et al. found salt content in salad to be significantly lower than the salt content in other meal components [27]. Finally, a general reduced amount of sodium in the food supply has been identified. In Denmark, salt content of industrial prepared bread sold from supermarkets was reduced during the years from 2009 to 2014 by approximately 10% on average [31]. Similarly, in the Netherlands, the salt content of bread, certain sauces, soups, potato crisps, and processed legumes and vegetables have been reduced over the period 2011–2016 [34].

Ready meals and foods consumed out of home make a significant contribution to our daily diets. Therefore, there is a strong need to develop further strategies to lower current salt intake levels as a public health tool [35]. A systematic review and meta-analysis found it was possible to reduce the salt content substantially in different kinds of reformulated food products without negative effect on consumer acceptability [36]. Janssen et al. found that consumption of reduced-sodium foods over a 3-week period (reduced by 29% to 61%) reduced daily sodium intake by 1 g, equaling 2.5 g salt, and was well-accepted by participants in an experimental real-life canteen setting [37]. Successful catering salt-reduction intervention has included various initiatives, such as restriction of food high in salt, menu modification, adding spices to develop additional flavor, salt removed from the tables, nutrition information on salt reduction to customers [38], education about the risk of high-sodium intake directed at cooks and restaurant owners and tips for lowering sodium contents [39], and implementation of a worksite canteen labelling scheme that resulted in a decrease in salt content of 0.25 g per 100 g [18].

The main strength of this study is the objective measurement of the actual sodium content of the foods eaten by the participants, using the duplicate portion technique and analyzing the nutritional content by an accredited laboratory. This method does not depend on the accuracy of self-report, food composition tables or standard recipes. Also, plate waste was taken into account and therefore the consumed food was measured instead of the served food. Further, the survey imposed a minimum response burden on the participants, resulting in a high response rate. Yet, it cannot be excluded that the participants may have changed their food choice behavior at lunch on account of their meal being documented. Another possible limitation was that salt added at the table was not included. Results therefore represent the efforts of the worksite canteen at one meal occasion, but do not reflect the total implication on salt intake of the customers. Also, the relatively small sample size makes it underpowered for exploration of differences between worksites and different customer profiles, and we do not know whether a change in sodium intake at lunch potentially influences salt intake at other meal occasions. Janssen et al., however, found that offering reduced-sodium foods at lunch did not trigger compensation behavior during the remainder of the day [37]. Finally, seven of the evaluated canteens in the present study were different from the study conducted 10 years before. However, analysis of data for the eight canteens that participated in both studies separately indicated the same trend in reduction in salt intake, thereby confirming the results found in the present study [40]. Also, although meals from different customers were sampled at the two measurement points no significant differences in participants’ characteristics with regard to gender distribution, age, BMI or occupation were seen between the two studies. Still, there may be other characteristics, which may have influenced the intake of salt. With regard to characteristic of the participating worksites, all of worksite canteens participating at the present study had buffet-style serving, compared with about half of the canteens at the study 10 years before [23]. The same tendency is seen in Danish worksite canteens in general, and Scourboutakos et al. argue that there has been a shift toward buffet-style dining halls in universities, in recent years, to give students greater flexibility and more food choices [41].

## 5. Conclusions

In conclusion, this study showed a gender difference in canteen lunch salt intake and further suggests a possible reduction in salt content of lunch meal consumed at Danish worksite canteens of 0.5 g per meal during a 10 years period. There is, however, a need for further reduction of salt content. The catering sector has a large role to play in helping consumers to reduce their salt consumption to meet the population target for salt intake. More evaluations of population-level interventions in real-world settings are needed, including sodium sources from food consumed outside of the home (e.g., restaurants and takeaways) to help understand and support more effective implementation of interventions to reduce salt intake further.

## Figures and Tables

**Table 1:** Characteristics of participating worksite canteens.

Characteristics	Worksite Canteens (*n* = 15)
Situated in urban settings (%)	53
Canteen outsourced (%)	33
Number of daily canteen lunch meals (Mean(range))	138 (40–400)
Occupation of the customers:	
Mainly sedentary job functions (>50%) (%)	67
Mainly male employees (>50%) (%)	60

**Table 2:** Characteristics of participants.

Characteristics	Participants (*n* = 240) (%)
Sex	
Women	41
Men	59
Age group (year)	
<30	16
30–39	26
40–49	33
>50	25
BMI	
<18	1
19–25	60
26–30	31
>30	8
Occupation	
Skilled	20
Unskilled	5
Office worker	68
Trainee and other	6

BMI: body mass index.

**Table 3:** Unadjusted and estimated mean salt intake for all participants.

Salt Intake	All (*n* = 240)		
	Unadjusted Mean	SD	Estimated Mean (95% CI)
g/meal	2.8	1.4	2.6 (2.2,3.0)
g/100 g	0.77	0.3	0.78 (0.69,0.88)
g/10 MJ	13.2	4.6	12.0 (10.6,13.6)

SD: standard deviation.

**Table 4:** Unadjusted and estimated mean salt intake according to gender and effect estimate of intake according to gender (adjusted for age, BMI, and occupation).

Salt Intake	Female (*n* = 99)			Male (*n* = 141)			Male-female	
	Un-adjusted Mean	SD	Estimated (95% Cl) Mean	Un-adjusted Mean	SD	Estimated (95% Cl) Mean	Estimated Median Difference (95% Cl)	*p*-Value ^1^
g/meal	2.4	1.1	2.2 (1.7,2.7)	3.2	1.5	2.9 (2.3,3.5)	0.8 (0.4,1.1)	**<0.001**
g/100 g	0.71	0.28	0.70 (0.59,0.83)	0.81	0.29	0.80 (0.68,0.93)	0.10 (0.02,0.17)	**0.011**
g/10 MJ	13.1	4.9	11.9 (10.0,13.8)	13.4	4.4	12.4 (10.6,14.4)	0.6 (-0.6,1.8)	0.325

^1^ Linear mixed model adjusted for age, BMI and occupation and random effects of worksite. Significant differences are highlighted in bold (*p* < 0.05).
